# Detection of Bladder Cancer Using Proteomic Profiling of Urine Sediments

**DOI:** 10.1371/journal.pone.0042452

**Published:** 2012-08-03

**Authors:** Tadeusz Majewski, Philippe E. Spiess, Jolanta Bondaruk, Peter Black, Charlotte Clarke, William Benedict, Colin P. Dinney, Herbert Barton Grossman, Kuang S. Tang, Bogdan Czerniak

**Affiliations:** 1 Department of Pathology, The University of Texas MD Anderson Cancer Center, Houston, Texas, United States of America; 2 Department of Urology, The University of Texas MD Anderson Cancer Center, Houston, Texas, United States of America; 3 Ciphergen Biosystems, Inc., Fremont, California, United States of America; 4 Department of Genitourinary Medical Oncology, The University of Texas MD Anderson Cancer Center, Houston, Texas, United States of America; 5 Department of Biostatistics & Applied Math, The University of Texas MD Anderson Cancer Center, Houston, Texas, United States of America; Queen Elizabeth Hospital, Hong Kong

## Abstract

We used protein expression profiles to develop a classification rule for the detection and prognostic assessment of bladder cancer in voided urine samples. Using the Ciphergen PBS II ProteinChip Reader, we analyzed the protein profiles of 18 pairs of samples of bladder tumor and adjacent urothelium tissue, a training set of 85 voided urine samples (32 controls and 53 bladder cancer), and a blinded testing set of 68 voided urine samples (33 controls and 35 bladder cancer). Using t-tests, we identified 473 peaks showing significant differential expression across different categories of paired bladder tumor and adjacent urothelial samples compared to normal urothelium. Then the intensities of those 473 peaks were examined in a training set of voided urine samples. Using this approach, we identified 41 protein peaks that were differentially expressed in both sets of samples. The expression pattern of the 41 protein peaks was used to classify the voided urine samples as malignant or benign. This approach yielded a sensitivity and specificity of 59% and 90%, respectively, on the training set and 80% and 100%, respectively, on the testing set. The proteomic classification rule performed with similar accuracy in low- and high-grade bladder carcinomas. In addition, we used hierarchical clustering with all 473 protein peaks on 65 benign voided urine samples, 88 samples from patients with clinically evident bladder cancer, and 127 samples from patients with a history of bladder cancer to classify the samples into Cluster A or B. The tumors in Cluster B were characterized by clinically aggressive behavior with significantly shorter metastasis-free and disease-specific survival.

## Introduction

Current pathogenetic concepts postulate that common neoplasms of the bladder arise in its epithelial lining (urothelium) via two distinct but somewhat overlapping pathways: the papillary and nonpapillary pathways. [1] Approximately 80% of the tumors that arise in the bladder are exophytic papillary lesions that originate from hyperplastic urothelial changes. They typically recur but usually do not invade the bladder wall or metastasize. The remaining 20% of bladder tumors are aggressive, nonpapillary carcinomas with a propensity for invading and metastasizing. Invasive bladder cancers typically occur in patients without a history of papillary tumors and originate from *in situ* preneoplastic lesions ranging from mild to moderate dysplasia (low-grade intraurothelial neoplasia, LGIN) to severe dysplasia and carcinoma *in situ* (high-grade intraurothelial neoplasia, HGIN). [2] The majority of aggressive high-grade non-papillary bladder carcinomas present at an advanced stage and necessitate chemotherapy and/or radical cystectomy to improve survival.

For studies of biomarkers, bladder carcinoma is an ideal disease model, because its development and progression can be monitored using noninvasive or minimally invasive techniques. [3] The mucosa of the bladder can be examined and biopsies can be obtained via an endoscopic procedure. In addition, the morphology of exfoliated urothelial cells and their constituents as well as secreted products can be scrutinized in urine at no risk to the patient.

Proteomic technologies that involve mass spectrometry coupled with ProteinChip Systems have been shown to facilitate the protein profiling of biological specimens. [4]–[6] The initial findings documenting the identification of serum and urine protein fingerprints for diagnosing several cancers [7]–[9] have been followed by reports raising concerns about problems with study design, reproducibility, calibration, and analytical procedures [10]–[13].

**Figure 1:**
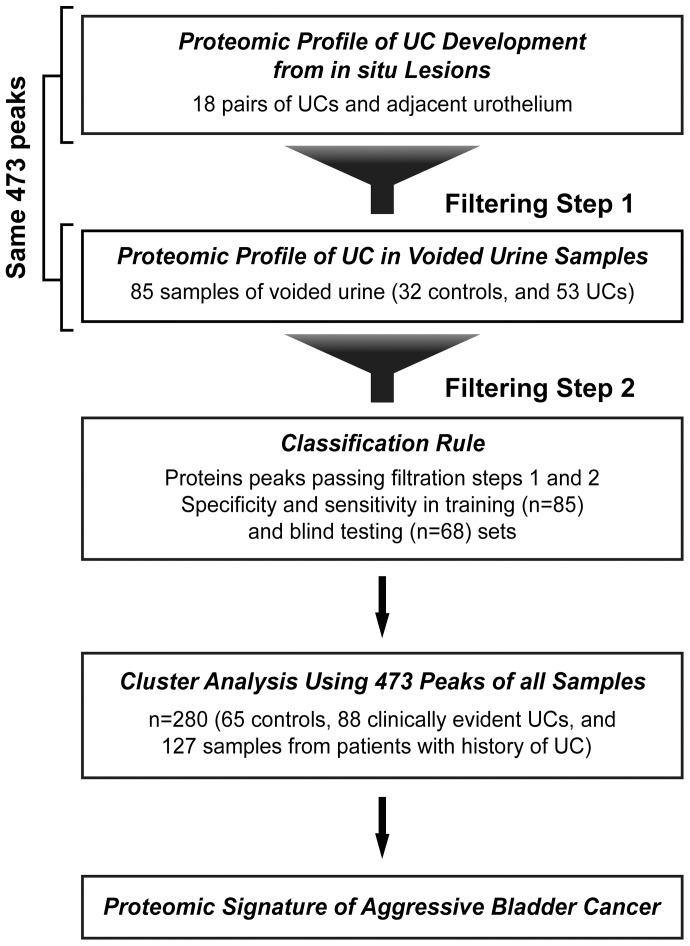
Analytical strategy used to develop classification rule for bladder cancer. The proteomic profile of bladder cancer development from *in situ* neoplasia was developed on a collection of proteomic spectra from paired samples of urothelial carcinoma (UC) and adjacent urothelium compared to normal urothelium. Using this approach, 473 protein peaks expressed in normal urothelium were identified. The same 473 were subsequently identified in the training set of voided urine samples from control subjects and patients with UC. The protein peaks first identified as abnormally expressed in tissue samples (filtering step 1) and then in the training set of voided urine samples (filtering step 2) were used to design a classification rule. The performance of the classification rule was assessed first in the training set and then in a blind testing set. Finally, a cluster analysis was performed using 473 protein peaks on all control and UC samples to identify the proteomic signature of aggressive bladder cancer.

This report outlines a strategy for protein profiling using surface-enhanced laser desorption and ionization time-of-flight (SELDI-TOF) mass spectroscopy to formulate a classification rule for detecting bladder cancer in voided urine samples and classifying clinically distinct classes of the disease.

**Figure 2:**
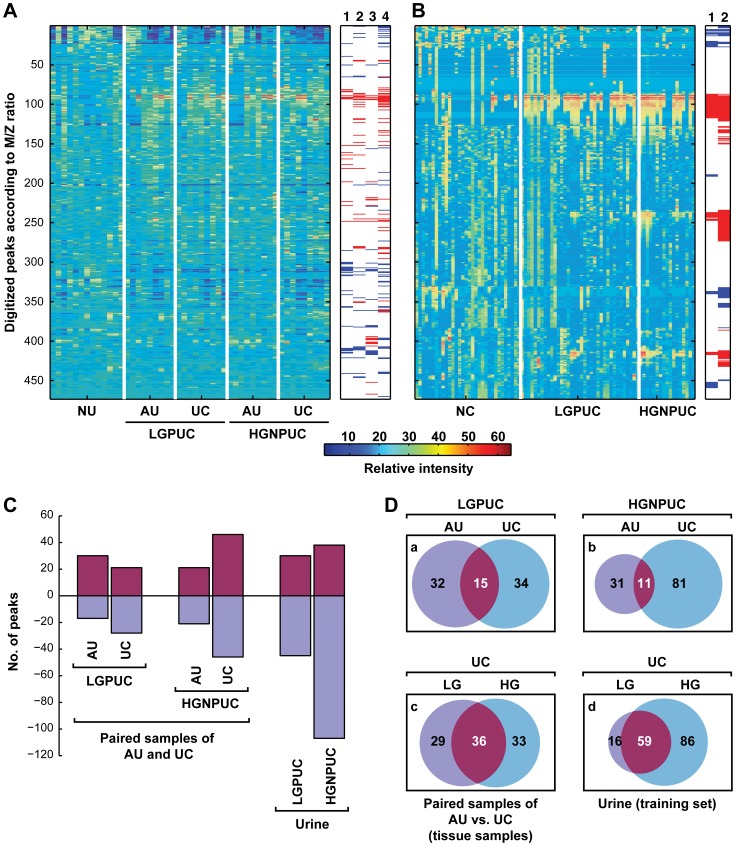
Proteomic profile of bladder cancer. (**A**) Digitalized proteomic profile of bladder cancer development from *in situ* neoplasia. Expression levels of protein peaks were analyzed on paired samples from adjacent urothelium (AU) and UCs compared to normal urothelium (NU). Each column represents UC or AU samples and each row corresponds to a digitalized protein peaks arranged according to *M/Z* ratios. Ratios of individual *M/Z* peak relative to NU are shown as a color saturation scale below the diagram. Samples corresponding to AU and UC are grouped according to their pathogenetic subsets representing low-grade (Grade 1–2) superficial papillary UC (LGPUC) and high grade (Grade 3) invasive UC (HGNPUC). The bar diagram on the right shows individual protein peaks with higher (red) and lower (blue) expression levels as compared to NU. Column 1: comparison between NU and AU LGPUC, 2: comparison between NU and LGPUC, 3: comparison between NU and AU HGNPUC, 4: comparison between NU and HGNPUC. (**B**) Proteomic profile of voided urine samples from control subjects (normal control, NC) and patients with UC dichotomized into LGPUC and HGNPUC categories. The bar diagram on the right shows individual protein peaks with higher (red) and lower (blue) expression levels as compared to NU. Column 1: comparison between NC and LGPUC; Column 2: comparison between NC and HGNPUC. (**C**) Number of protein peaks with higher (maroon) and lower (purple) expression levels as compared to NU identified in paired tissue samples of AU and in voided urine samples of patients with UC compared to NC. (**D**) Proportion of proteins peaks with similar and dissimilar expression pattern.

## Methods

### Tumor and Urine Samples

All human tissues were collected wpith written informed consent under protocols approved by the M. D. Anderson Institutional Review Board and the samples were analyzed anonymously. We analyzed the protein expression profiles of 18 pairs of samples of bladder tumor and adjacent urothelium tissue, 88 voided urine samples from patients with clinically evident bladder cancer, and 127 voided urine samples from patients with a history of bladder cancer (HiUC) and no cystoscopic or pathologic evidence (negative bladder biopsy and/or voided urine cytology) of bladder cancer at the time of urine collection. For paired samples of adjacent urothelium and bladder tumor tissue, we obtained baseline protein profiles from urothelial cell suspensions of 13 ureters with no evidence of urothelial neoplasia removed during nephrectomy for renal cell carcinoma. For urine samples, we obtained baseline protein profiles from 65 healthy individuals. The profiles were initially analyzed in paired samples of adjacent urothelium and bladder tumor tissue. They were then compared to the profiles identified in the initial 85 samples of urine (32 controls and 53 bladder cancer) referred to as the training set. Subsequently, the proteins that were significantly up- or down-regulated in both sets were used in a diagnostic algorithm first on the training set (n = 85) of urine samples and then on a blinded testing set (n = 68; 33 controls and 35 bladder cancer). Finally, the proteomic profiles of all samples (65 normal controls, 88 bladder cancers, and 127 HiUCs) were analyzed using unsupervised clustering.

**Figure 3:**
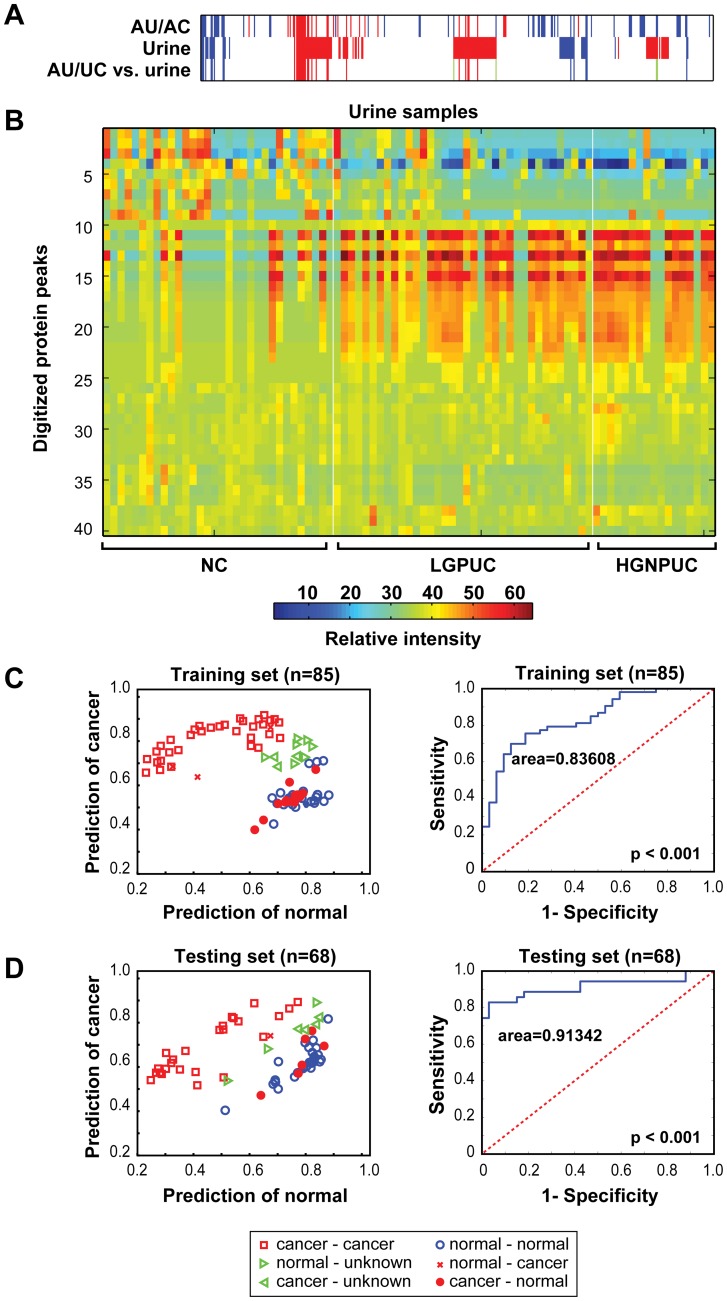
Detection of bladder cancer by proteomic profiling using 41 protein peaks. (**A**) Up regulated (red) and down regulated (blue) protein peaks identified in AU and UC (upper row) and voided urine samples (mid row) and the protein peaks consistently found in both sets of samples (lower row). (**B**) Heat map for 41 protein peaks identified by filtering step 2. (See Figure 1) (**C**) Classification of individual samples (left panel) and ROC curve (right panel) in the training set. (**D**) Classification of individual samples (left panel) and ROC curve (right panel) in the testing set.

The intraurothelial precursor conditions were classified on parallel sections from areas of adjacent mucosa as LGIN or HGIN. [2] The presence of normal, dysplastic, or malignant cells in scrapings from adjacent urothelium tissue was confirmed using microscopic evaluation of cytospin preparations. The tumors were classified according to the three-tiered World Health Organization histologic grading system and their growth patterns (papillary versus nonpapillary). [14] The depth of invasion was recorded according to the TNM (tumor–node–metastasis) staging system. [15] Stage T_1_ (lamina propria invasion) has been divided into T_1a_ (no muscularis mucosae invasion) and T_1b_ (muscularis mucosae invasion), which has a significantly higher risk of progression. [16] The tumors were dichotomized into superficial (T_a_-T_1a_) and invasive (T_1b_ and higher) groups, as previously described. [17]

**Figure 4:**
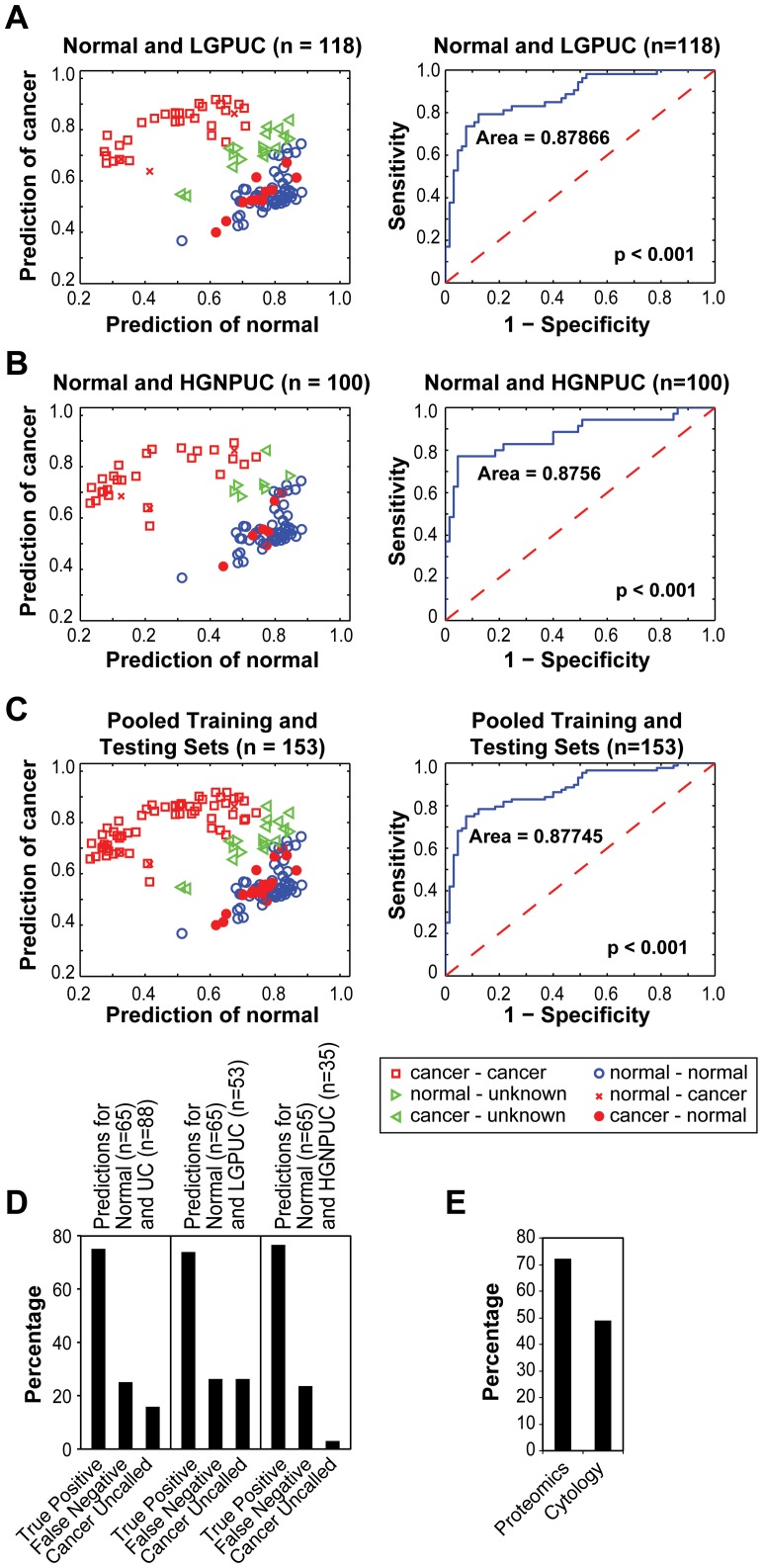
Detection of bladder cancer by proteomic profiling using 41 protein peaks in LGPUC and HGNPUC. (**A**) Classification of individual samples (left panel) and ROC curve (right panel) based on 65 benign control samples and 53 samples from patients with LGPUC. (**B**) Classificatin of individual samples (left panel) and ROC curve (right panel) based 65 benign control samples 35 samples from patients with HGNPUC. (**C**) Classification of individual samples (left panel) and ROC curve (right panel) based on 65 benign control samples and 88 samples from patients with UC.(combined training and testing sets) (**D**) Comparison of diagnostic accuracy of proteomics and cytology on 39 samples from patients with UC. (**E**) Classification of individual samples by proteomics based on the combined testing and training sets as well as for LGPUC and HGNPUC separately.

Cell suspensions from adjacent urothelium and bladder tumor tissue were prepared as described previously. [16] In brief, cystectomy samples of previously untreated urothelial carcinomas were used after obtaining informed consent from the patients. Each cystectomy sample was opened longitudinally along the anterior wall of the bladder and pinned down to a paraffin block. One representative section from the central area of grossly identified tumor was obtained for proteomic profiling. The presence of tumor in the tissue was confirmed via analysis of frozen sections. To minimize contamination with nontumor tissue, we dissected an area of tumor tissue from the frozen block. We prepared urothelial cell suspensions from adjacent urothelium tissue by scraping the mucosal surface. The purity of the samples was determined via cytologic examination of the cytospin preparations. Only the samples that yielded more than 90% microscopically intact normal, dysplastic, or malignant urothelial cells were used for protein analysis. For processing, the cells were transferred to conical tubes containing phosphate-buffered saline (PBS). The frozen tumor tissue was transferred to a similar conical tube containing PBS, which was mechanically agitated to release tumor cells. Before preparing cell lysates, we precleaned the cell suspensions via Ficoll Histopague-1077j (Sigma Diagnostics, Inc. St. Louis, MO, USA) gradient centrifugation. For storage, the cell pellets were resuspended in PBS containing 20% dimethyl sulfoxide and frozen in liquid nitrogen. Voided urine samples were treated in the same manner. [3]

**Figure 5:**
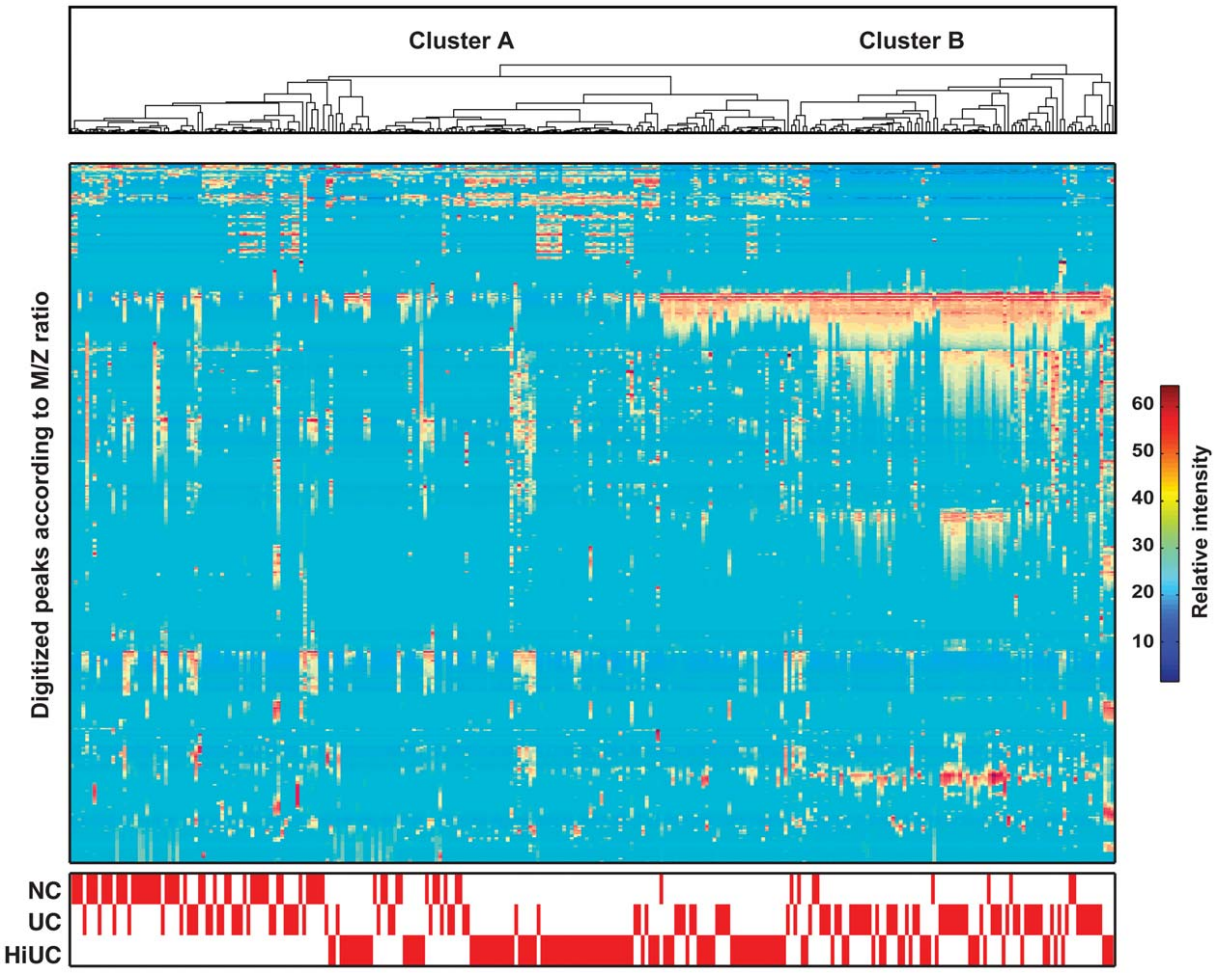
Unsupervised hierarchical clustering of voided urine samples (n = 280). The cohort included 65 normal controls (NC), 88 patients with clinically evident bladder cancer (UC) and 127 patients with a history of bladder cancer (HiUC). Clustering was performed using Euclidean distance and the matrix of expression intensities for 473 protein peaks. Each column represents a voided urine sample and each row corresponds to the digitalized protein peaks arranged according to *M/Z* ratios.

Processing of the urine samples was completed within 1–4 hours of receipt. The volume of urine ranged from 10 to 50 ml. Urine samples were centrifuged at 2500 rpm for 10 minutes at room temperature. The cell pellet was resuspended in 2 ml Dulbecco’s modified Eagle’s medium (DMEM). The new conical tube (50 ml) was filled with 20 ml DMEM, and 5 ml Ficoll was placed in the bottom. The urinary cells were then transferred to the top of the solution. After centrifugation at 2500 rpm for 20 minutes at room temperature, the 10-ml upper layer was removed, and the interface (∼8 ml) with urinary cells was transferred to a new conical tube (25 ml). The sample was centrifuged again at 2500 rpm for 10 minutes at 4°C. Finally the cells were collected, resuspended in 2 ml of DMEM with 10% dimethyl sulfoxide, and stored at −80°C for later use.

**Figure 6:**
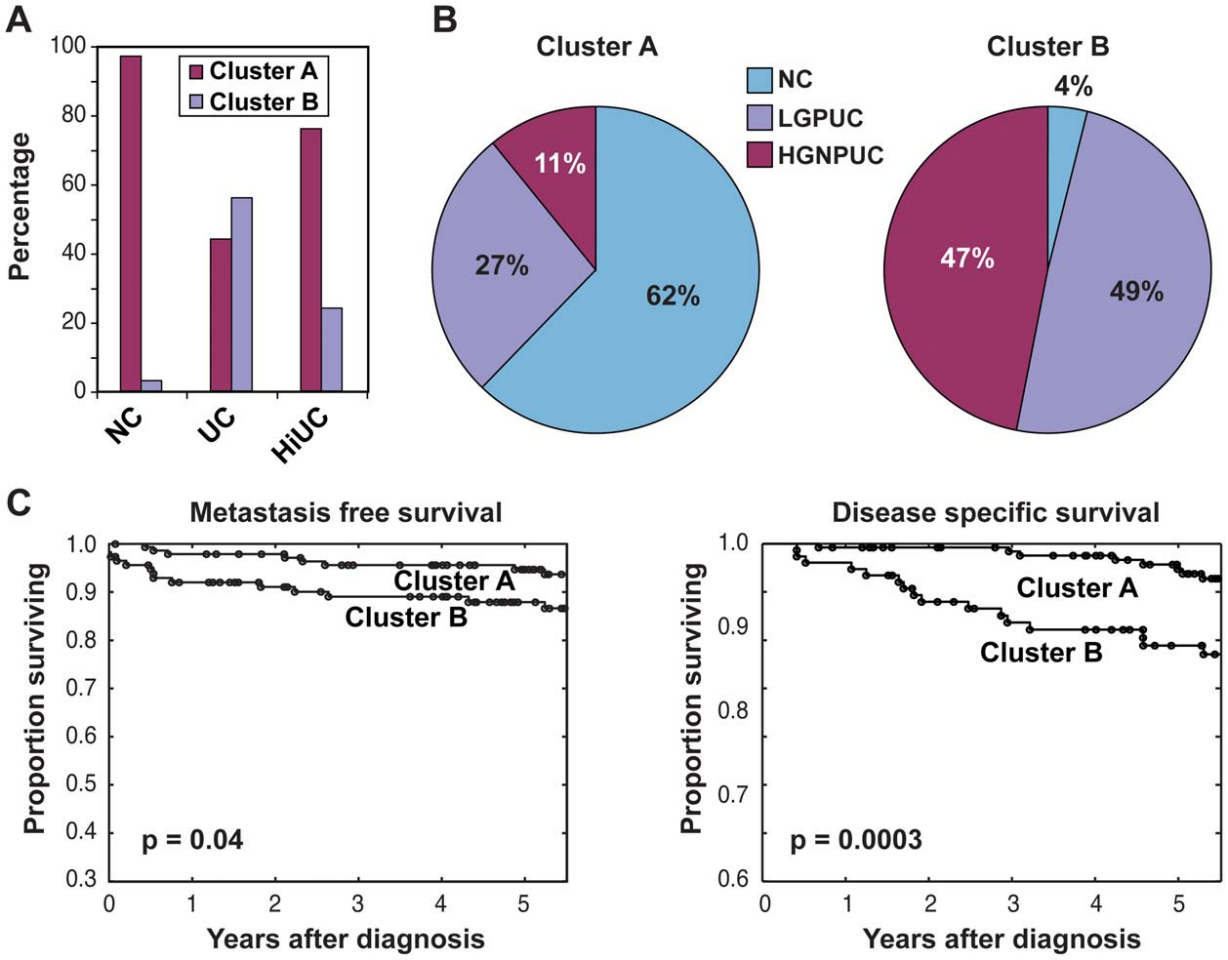
Distribution of various samples in clusters A and B. (**A**) Distribution of voided urine samples in cluster A and B of normal controls (NC), patients with clinically evident bladder cancer (UC) and patients with history of bladder cancer (HiUC). (B) Distribution of voided urine samples in Clusters A and B according to histologic grade and stage dichotomized into low grade invasive superficial papillary UC (LGPUC, pT_a_ – pT_1a_) and high grade non-papillary UC (HGNPUC, T_1b_ and higher). (**C**) Kaplan – Mayer plots of metastasis and disease specific survival of patients with bladder cancer in Clusters A and B.

### Preparation of Cell Lysates and Proteomic Analysis

Cell lysates were prepared as referenced in the Bio-Rad Web site. [18] Briefly, the samples were towed, centrifuged at 5000 g for 10 minutes, washed in PBS, and resuspended in a lysis buffer (10 mM Tris [pH = 9], 10 mM NaCl, 0.1% dodecyl mattoside). The protein lysates were prepared via sonication using a probe sonicator (Cole-Parmer Instrument Co., Chicago, IL, USA) set at 5 watts 10 times for 15 seconds with 45-second intervals of cooling on ice. Total protein content was measured in each sample using a Micro BCA protein assay reagent kit (Pierce, Rockford, IL, USA). Immobilized metal affinity IMAC3 capture chips (Ciphergen Biosystems, Fremont, CA, USA) were used for proteomic analysis. Chips activated by copper sulfate were briefly washed in deionized water and incubated with 100 mM sodium acetate (pH, 4.5) for 5 minutes to remove any excess Cu^+2^ and again washed with deionized water. The chips were briefly equilibrated with cell lysis buffer and incubated for 1 hour with protein lysates containing 1 µg of total protein in a volume of 3–8 µl of lysis buffer. Before reading, the chips were washed three times with lysis buffer, two times with deionized water, air-dried, and crystalized with 0.3 µl of sinapinic acid in 50% acetonitrile/1% trifluoroacetic acid. All preparatory steps were carried out at room temperature. The protein profiles were analyzed using a Ciphergen PBS II ProteinChip Reader (Ciphergen Biosystems Fremont, CA, USA). Before each series of measurements, the system was calibrated using an “all-in-1” standard from Ciphergen Biosystems, and the proteomic profile of a normal reference tissue, i.e., of normal urothelium tissue or of the urine sediment of normal individuals, was tested.

**Figure 7:**
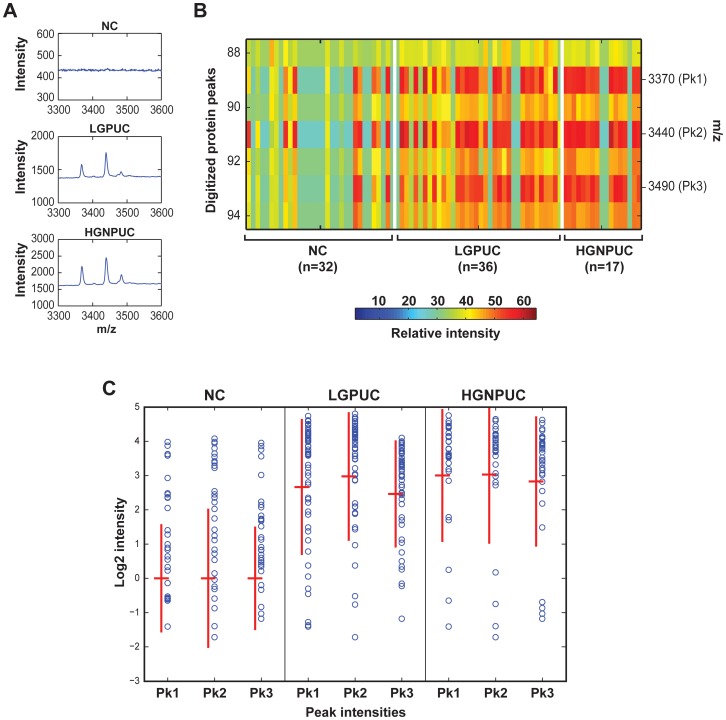
Expression patterns of selected protein peaks with molecular weights corresponding to α-defensins. (**A**) Protein profiles between 3300 and 3600 m/z of representative samples corresponding to NC, LGPUC and HGNPUC showing the expression pattern of three protein peaks with 3370, 3440 and 3490±10 m/z referred to as peaks 1–3 (pk 1–3) representing the cluster of α-defensins. (**B**) Zoomed heat map showing the expression pattern of α-defensin cluster in the training set. (**C**) The expression intensities of α-defensin cluster corresponding to pk 1–3 in pulled samples of testing and training sets of NC, LGPUC and HGNPUC. Crossed red lines and vertical bars represent mean and standard deviations. Two sample T-test was used to compare the log2-transformed peak intensities in cancer samples and controls for each respective peak (p<0.001).

To assess the accuracy of the measurements, we performed several studies. [19] We evaluated the intensities of 26 peaks in 24 replicate spectra of the same sample from normal urothelium tissue to check reproducibility **(Figure S1)**. Peak coefficients of variation (CVs) ranged from 13.7% to 63.1% of the mean. The median CV was 22.7%, and the interquartile range was from 19.4% to 27.7%. We also tested the sensitivity of the mass-to-charge ratio (M/Z) values to an amount of the total protein by varying loadings from 0.5 to 2 µg, and the M/Z readings varied by less than 1% (data not shown).

### Analytical Procedures

All spectra were exported as *.xml files using Ciphergen software. The raw spectra were processed using MATLAB scripts developed in-house to (a) remove random noise, (b) subtract the low-frequency baseline, and (c) detect and quantify individual sample peaks. Denoising and baseline subtraction were performed using the wavelet thresholding approach. [20] After denoising, the spectra were normalized. Peak detection made use of the mean spectrum after denoising. [21] The data from all the spectra were summarized in a matrix of peak intensities, with each row corresponding to a specific *M/Z* value (a peak) and each column corresponding to a specific sample.

Classification accuracy was assessed via sensitivity and specificity and by positive and negative predictive values. The classification rule was also assessed using receiver operating characteristic (ROC) curves. The varying parameter in the ROC curve was the angle between the decision boundary line and the normal X axis: an angle of 0° led to all samples being classified as normal, and an angle of 90° led to all samples being classified as cancer. In addition, voided urine spectra were examined using unsupervised clustering to assess the degree of the associations between the two clusters and various clinicopathologic covariates, including follow-up.

## Results

The analytical strategy used in our study to formulate a protein profile for detecting bladder cancer is summarized in **Figure 1**. To identify the optimal combination of protein peaks diagnostic of bladder cancer, we first analyzed a proteomic profile of its development from *in situ* neoplasia and compared it to the proteomic profile of voided urine sediment from bladder cancer patients. To identify the proteins that were abnormally expressed during early bladder cancer development, we analyzed the patterns of their expression in 18 paired samples of bladder tumor and adjacent urothelium tissue and compared them to their expression pattern in 13 samples of normal urothelium. We first selected peaks that were clearly identifiable in tissue samples and used t-tests to identify peaks that had significant differential expression across different categories of paired bladder tumor and adjacent urothelial samples. Using this approach, referred to as filtration step 1, we identified 473 protein peaks expressed in normal urothelium tissue and sets of up- and down-regulated proteins, which were somewhat overlapping but distinct, thereby signifying the development of bladder cancer from *in situ* neoplasia via papillary and nonpapillary pathways. Since voided urine sediments may contain a mixture of tumor and nontumor cells, including inflammatory, stromal, and peripheral blood cells as well as necrotic cells with degenerated proteins, we focused on the same 473 peaks identified in the tissue samples and examined their intensities in a training set of voided urine samples from 53 patients with clinically evident bladder cancer and 32 healthy individuals. In this phase, referred to as filtration step 2, we searched, again using t-tests, for peaks with significant differential expression between cancers and controls.

Examples of SELDI-TOF spectra from samples of normal urothelium tissue and paired samples of adjacent urothelium and tumor tissue as well as the results of filtration step 1 are shown in **Figure 2A**. The spectra from voided urine sediments of bladder cancer patients and normal controls and the results of filtration step 2 are shown in **Figure 2B**. The differences in the protein expression profiles of tumors identified in tissue and voided urine samples are summarized in **Figures 2C and D**. It is evident that HGINs or high-grade nonpapillary urothelial carcinomas (HGNPUC) have somewhat overlapping but distinct protein expression patterns that can be also identified in adjacent urothelium tissue. This finding implies that abnormal protein expression profiles can be identified in surface urothelium tissue before the development of clinically evident cancer. By comparing the patterns of abnormally expressed proteins in bladder tumors, their adjacent urothelia, and voided urine samples from the training set, we identified a set of distinct up- and down-regulated proteins that were present in both bladder tumor tissues and voided urine sediment samples of patients with bladder cancer that was retained after both filtration steps. (**Figures 3A and B**).

Using only the peaks that passed both filtration steps, we used the matrix of 41 protein peak intensities to construct a classification rule for individual samples in the training and testing sets. (**Figure 3C**) The positions of individual samples in relation to the X (normal) and Y (cancer) axis were defined using a pair of numbers indicating their associations with both normal and cancer protein profiles. In this classification rule, samples with high associations with normal protein profiles and low associations with cancer profiles were clustered in region 1 and were classified as benign. In contrast, samples with low associations with normal profiles and high associations with cancer profiles were clustered in region 2 and were classified as cancer. Samples with equally weak or strong associations with normal and cancer profiles formed were clustered in region 3 and were designated as ambiguous. The boundaries of these clusters were defined using leave-one-out cross-validation.

Classification accuracy was initially assessed on the training set in terms of sensitivity of 0.59, specificity of 0.90, positive predictive value within the training set of 0.92, negative predictive value within the training set of 0.53, and ROC curve area of 0.84. (**Figure 3D**) Having defined the classification rule on the training set, we then validated its accuracy on the blinded testing set of 33 normal control samples and 35 bladder cancer samples, which yielded a sensitivity of 0.80, specificity of 1.0, positive predictive value on the testing set of 1.0, negative predictive value on the testing set of 0.83, and ROC curve area of 0.91. (**Figure 3D**) Cases that were deemed ambiguous were excluded when computing sensitivity, specificity, positive predictive value, and negative predictive value. All cases were retained for fitting ROC curves.

To assess how the classification rule based on the matrix of 41 protein peak intensities performed in different subsets of bladder cancer, we combined the training and testing sets and assessed its diagnostic accuracy for low-grade papillary urothelial carcinoma (LGPUC) and HGNPUC separately. Analysis of 65 benign control samples and 53 LGPUC samples yielded a sensitivity of 0.74, specificity of 0.95, positive predictive value of 0.91, negative predictive value of 0.84, and ROC curve area of 0.88. **(** **Figure 4A** **)** Similar analysis of 65 benign control samples and 35 HGNPUC samples yielded a sensitivity of 0.77, specificity of 0.95, positive predictive value of 0.90, negative predictive value of 0.88, and ROC curve area of 0.88. **(** **Figure 4B** **)** Analysis of the overall classification accuracy for the combined training and testing sets yielded a sensitivity of 0.75, specificity of 0.95, positive predictive value of 0.95, negative predictive value of 0.75, and ROC curve area of 0.88. **(** **Figures 4C and D** **)** Analysis of 39 samples from patients with bladder cancer for which the parallel data on the results of voided urine cytology were available indicated that classification based on the proteomic data correctly diagnosed 28 (72%) samples, whereas voided urine cytology correctly diagnosed 19 (49%) samples. **(** **Figure 4E** **)** Testing the difference between positive samples identified by proteomics and cytology using a z-test for proportions yielded a two-sided p value of 0.032.

Unsupervised clustering was carried out using Euclidean distance and complete linkage on all 65 normal control samples, 88 samples from patients with clinically evident bladder cancer, and 127 samples from patients with a HiUC. (**Figure 5**) Using the matrix of expression intensities for all 473 protein peaks, we classified the samples into two major groups. The first group (cluster A) consisted of a majority (97%) of the benign control samples. (**Figure 6A**) The second group (cluster B) consisted of 56% of the samples from patients with clinically evident bladder cancer. Interestingly, only 24% of the samples from patients with a HiUC co-segregated with samples from patients with clinically evident bladder cancer in cluster B. The remaining 76% of the samples from patients with a HiUC and 44% of the samples from patients with clinically evident bladder cancer co-segregated with benign control samples in cluster A. We hypothesized that this co-segregation may signify distinct classes of bladder cancer and analyzed the pathologic and clinical parameters of the samples in clusters A and B. **(** **Figure 6B** **)** Cluster A comprised predominantly normal control samples (62%) in addition to 27% LGPUC and 11% HGNPUC. In contrast, cluster B comprised only 4% normal control samples, 49% LGPUC, and 47% HGNPUC. The tumors in cluster B were characterized by significantly shorter metastasis-free and disease-specific survival than tumors from cluster A. (**Figures 6C and D**) Overall, the probability of dying of bladder cancer for patients in cluster B was approximately 12%, whereas the probability of dying for those in cluster A was less than 5%.

Although we did not perform the identification of the peaks used in a classification rule, we address their potential nature by focusing on the three most prominent peaks used in the analysis of our protein expression profiles. The cluster of three protein peaks with m/z values most likely corresponding to α-defensins was included in the classification rule. [22]–[24] The examples of SELDI-TOF spectra profiles between 3300 and 3600 m/z in representative urine samples of negative control, LGPUC and HGNPUC depicting the expression pattern of three peaks corresponding to α-defensins and the zoomed heat map in the training set are shown in **Figure 7A and B** **.** The expression pattern of the same proteins in the combined training and testing sets shows their overexpression in LGPUC and HGNPUC. **(** **Figure 7C** **)** The overexpression pattern of α-defensins is highly significant in both LGPUC and HGNPUC as compared to normal controls and even if taken out of context of the 41 anonymous protein peaks used in the classification rule, these proteins perform reasonably well as diagnostic markers (sensitivity 0.77 and specificity 0.84). **(** **Figure 7C** **)**.

## Discussion

The study design for proteomic profiling typically consists of a comparison of proteomic patterns of samples from patients with cancer and benign control samples using artificial intelligence algorithms such as genetic algorithms or tree analysis. [25]–[29] Such an approach identifies a limited number of anonymous protein peaks for discriminating cancer from benign tissue. When such peaks were identified by peptide sequencing, they represented, in general, the so-called acute phase proteins rather than tumor-specific products [28].

Several studies using proteomic profiling of voided urine for bladder cancer detection with SELDI platform were recently published. [30], [31] These studies used various approaches to protein spectra analysis that range from the use of artificial intelligence algorithms combined with supervised clustering to individual peak and peak cluster identification as diagnostic discriminatory parameters. [30], [31] As expected, the automatic clustering algorithm segregated controls from cancer samples with high sensitivity (80%) and specificity (>90%) in the training set but was associated with a dramatic drop of both sensitivity and specificity in the testing set to a range of approximately 50% and 60% respectively. [30] The combinatorial approach of individual biomarkers and biomarker clusters provided sensitivity of 87% and specificity of 66% but this study did not include separate training and testing sets of samples. [31] Interestingly, the individual markers identified by this approach included the peaks corresponding to α-defensin family. Proteomic studies for diagnosis and prognosis of solid human malignancies including bladder cancer are based on serum or voided urine analysis and involve wide-range of technologies. The technologies used in biomarker design range from SELDI/MALDI-TOF MS through liquid chromatography or capillary electrophoresis mass spectroscopy and gel-based approaches to protein arrays. [32], [33] More recent metabolic approaches combined several techniques such as high performance liquid chromatography (HPLC) with gas chromatography or mass spectroscopy (MS) for the analysis of human urine metabolites in search for diagnostic, prognostic, and therapy monitoring biomarkers. [34]–[36] These studies support the potential for proteomic profiling as a non-invasive tool for detecting and monitoring bladder cancer. Recent studies of the SELDI-TOF approach reported good reproducibility of peak intensities and stability of M/Z reading ratios among five participating laboratories when the same sample preparation protocol and analytical formula were used. [37] Our multi-laboratory reproducibility study revealed similar variability of peak intensities within the range of the median CV of approximately 20% and good stability of M/Z reading ratios, which varied by less than 1%. **(Figure S1)**.

The approach outlined in our study was designed after multiple attempts to identify protein fingerprints for diagnosing bladder cancer by comparing the protein profiles of voided urine from cancer patients with those of benign controls. In general, the attempts using artificial intelligence algorithms produced satisfactory results in the initial training set and failed in the second blind dataset, often performing no better than chance. The critical components of the successful strategy presented in this report were (1) the generation of proteomic profiles of bladder cancer development from *in situ* neoplasia, (2) the identification of the same 473 protein peaks in tissue and voided urine samples, and (3) the use of two filtration steps for identifying 41 protein peaks that were differentially expressed in cancer patients and controls that could be identified in both tissue and voided urine. The matrix of these 41 protein intensities was used to define a classification rule, which detected cancer with a high degree of sensitivity and specificity in both training and blind testing sets. The proteomic classification rule performed with similar accuracy for LGPUC and HGNPUC. Moreover, the preliminary data indicated that proteomics may be more efficient in diagnosing bladder cancer than conventional voided urine cytology, but this finding must be verified in a larger independent sample set. The limited analysis of the diagnostic formula comprising 41 peaks and cytology on 39 samples indicate that the sensitivity of proteomics (72%) is significantly higher than voided urine cytology (49%). The recently published data comparing urine cytology and other biomarker tests, such as NMP22 and UroVysion FISH indicate high specificity (>90%) and low sensitivity (<30%) of cytology. [38] Although cytology is quite specific and sensitive for a high grade variant of urothelial carcinoma, it is considered to be inefficient for the detection of low grade urothelial tumors. [22], [39]–[41] By comparing the performance of our proteomic diagnostic formula with cytology we show that proteomics may perform equally well in both high grade and low grade urothelial carcinomas. Therefore, combining proteomic profiling with other diagnostic modalities including cytology may improve the detection of especially low grade urothelial tumors.

Unsupervised clustering using all 473 proteins identified clinically distinct subsets of bladder cancer corresponding to indolent and aggressive variants of the disease. In general, the proteomic profiles from voided urine sediments of patients with bladder cancer that clustered with benign controls were indicative of a better prognosis, with longer metastasis-free and disease-free survival, than samples from patients with bladder cancer that formed a distinct cluster.

Our study of proteomic expression profiles concerns 473 anonymous protein peaks and 41 of them were used to construct a classification rule. The true nature of these peaks is unknown but as evidenced by prior studies the proteomic profiling of body fluids, including urine from cancer patients, typically do not identify oncogenic or tumor suppressor-like proteins. Most of the peaks in such profiles correspond to so-called acute phase proteins responsible for immune responses which are unlikely directly involved in tumor development. [42]–[46] The three most prominent proteins included in our analytic formula, as well as in the analysis of the global proteomic profiles, correspond by their molecular mass to such proteins and most likely represent α-defensins. [31], [47]–[49] Defensins are involved in tissue specific regulation of inflammation but they were also documented as playing a role in tumor related cellular activities such as apoptosis and transcriptional regulation. [50] Their overexpression has been documented in several human malignancies including bladder cancer and was shown to be associated with tumor invasiveness. [23], [32], [49] Therefore, progressive deregulation of protein expression patterns in voided urine samples of patients with bladder cancer may be observed in aggressive variants of the disease. This may explain the relationship between global expression patterns of proteins in voided urine and clinical aggressiveness as identified by unsupervised clustering using the matrix of 473 protein peaks.

In summary, the analytical strategy described in this study facilitates the identification of protein expression peaks for diagnosis and prognosis of bladder cancer. The differences in protein expression profiles can be identified in voided urine samples of patients with bladder cancer compared to benign controls and in patients with low- versus high-grade bladder cancer and could be used as a noninvasive method for detecting and monitoring bladder cancer.

## Supporting Information

Figure S1 **Reproducibility of SELDI spectra.** Intensity values for 26 peaks in each of 24 replicate spectra.
(TIF)
