# Supplementary figures and images for: Detection of Bladder Cancer Using Proteomic Profiling of Urine Sediments
Source: PLoS One. 2012 Aug 3;7(8):e42452. doi: 10.1371/journal.pone.0042452 (PMC3411788; doi:10.1371/journal.pone.0042452)

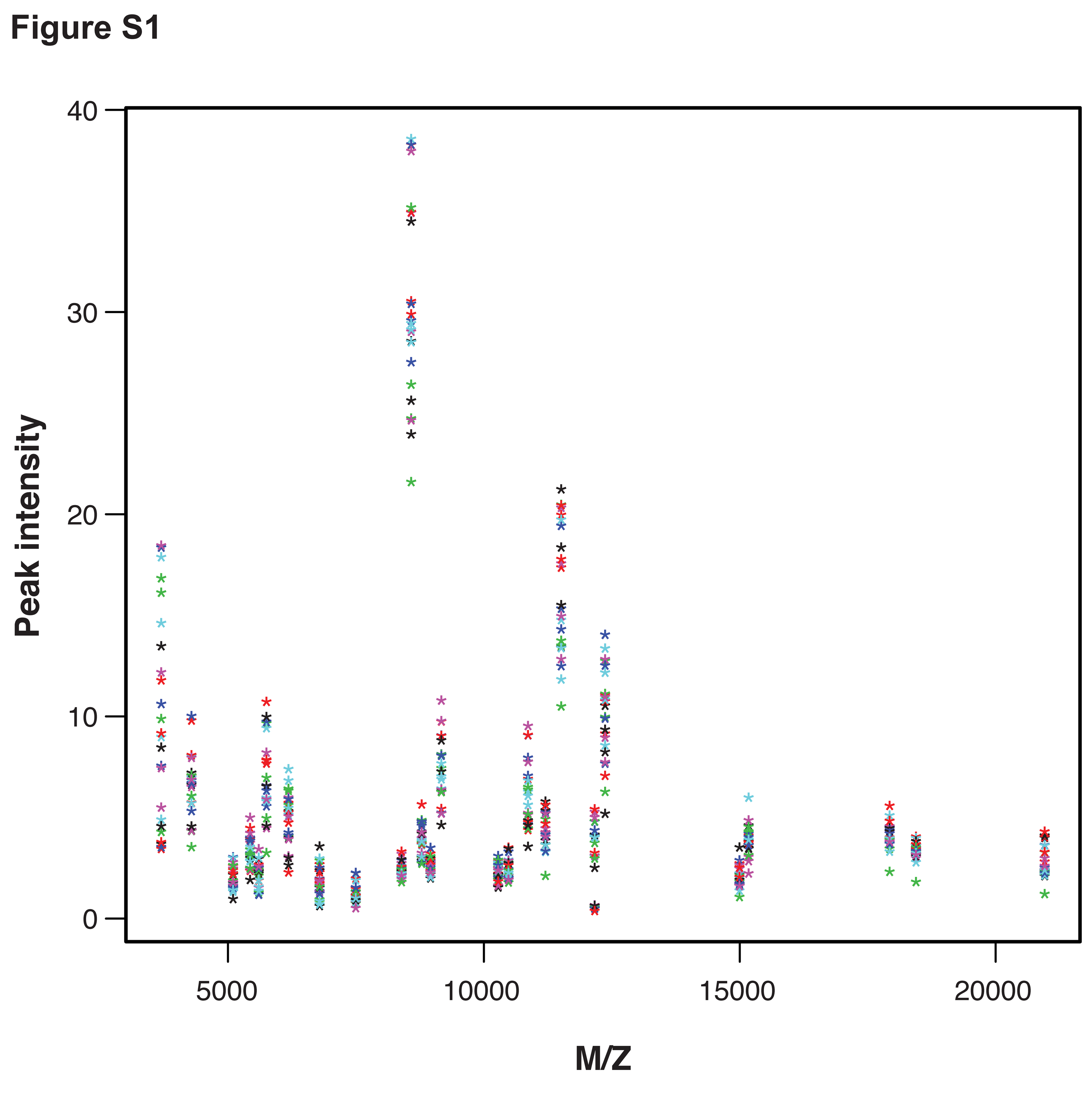

Supplement: Figure S1 — Reproducibility of SELDI spectra. Intensity values for 26 peaks in each of 24 replicate spectra. (TIF) [file pone.0042452.s001.tif]
